# Association of anemia and renal function test among diabetes mellitus patients attending Fenote Selam Hospital, West Gojam, Northwest Ethiopia: a cross sectional study

**DOI:** 10.1186/2052-1839-13-6

**Published:** 2013-05-07

**Authors:** Alemayehu Abate, Wubet Birhan, Abebe Alemu

**Affiliations:** 1 Bahir-Dar regional laboratory, Bahire Dar, Amahara Regional State, Ethiopia; 2 College of medicine health sciences, School of biomedical and laboratory sciences, Department of clinical chemistry, University of Gondar, Gondar, Ethiopia; 3 College of medicine health sciences, School of biomedical and laboratory sciences, Department of medical Parasitology, University of Gondar, Gondar, Ethiopia

## Abstract

**Background:**

Anemia is a common problem in diabetic patients. Diabetic patients have a greater severity of anemia as the level of Glomerular Filtration Rate (GFR) decreases compared to non-diabetic patients. Despite these facts, anemia is unrecognized and largely untreated in patients with diabetes in Ethiopia particularly in those patients attending Fenote Selam Hospital. Therefore, this study was aimed to assess the association of anemia and renal function test among diabetes mellitus patients attending Fenote Selam Hospital, North West of Ethiopia.

**Methods:**

An Institutional -based cross-sectional study was conducted from February 2012 to April 2012 on diabetes mellitus (DM) patients. Systematic random sampling technique was used to get the total sample size of 384 patients. A total of seven ml of venous blood was collected from diabetes mellitus patients; two ml was collected by EDTA anticoagualted vacutainer test tube for haemoglobin determination and 5 ml venous blood was collected by plain vacutainer tube for creatinine and Blood urea nitrogen determination. The data were double entered and analyzed using SPSS-16 statistical software. The degree of association between independent and dependent variables was assessed using bivariate and multivariate logistic regression analysis in terms of P-value and odds ratio with 95% confidence interval.

**Results:**

Out of the total 384 DM patients included in the study 73 (19%) were anemic. Fifty three (13.8%), forty eight (12.5%), and two hundred eighty three (73.7%) DM patients had an estimated GFR <60 ml/min/1.73 m, 60 – 90 ml/min/ 1.73 m, and > 90 ml/min/1.73 m respectively. One hundred eleven (28.9%) diabetic patients had increased urine albumin level. There was a statistically significant association between anaemia and Glomerular filtration rate (P<0.05) with Odds ratio of 8.58 and CI (10.21, 49.94). As the glomerular filtration rate increase, the risk to be anemic will decrease dramatically.

**Conclusion:**

The study showed that there was a significant association between anaemia and Glomerular filtration rate in DM patients. Therefore, DM patients should be strictly monitored for renal failure and anemia for proper management of diabetes patients.

## Background

Anemia is defined by World Health Organization (WHO) criteria: < 13 g⁄ dl for men and < 12 g⁄dl for women [1]. The etiology of anemia in diabetes is multifactorial and includes inflammation, nutritional deficiencies, concomitant autoimmune diseases, drugs, and hormonal changes in addition to kidney disease. Anemia that is associated with erythropoietin deficiency may have prognostic significance for persons with nephropathy [2].

Anemia is associated with an increased risk of the vascular complications of diabetes including nephropathy, retinopathy, neuropathy, impaired wound healing, and macrovascular disease [3]. The strong link between the kidney and anemia in diabetes probably reflect the unique vulnerability of the renal microcirculation to damage in diabetes [4]. Moreover, it is likely that significant damage is present before albumin is found in the urine.

Anemia occurs earlier, and is more severe, in chronic kidney disease (CKD) related to diabetes than in non-diabetic CKD [5]. Patients presenting with diabetic nephropathy commonly have a greater degree of anemia for their degree of renal impairment than those presenting with other causes of renal failure, and anemia develops earlier in these patients than in those with renal impairment from other causes [6].

A number of studies have reported the prevalence of anemia in people with diabetes and have suggested that up to 25% have previously unrecognized anemia [6,7]. Recent studies have identified anemia as a risk factor for the need for renal replacement therapy in diabetes; in addition, a lower HGB is significantly associated with a more rapid decline in the glomerular filtration rate (GFR). Furthermore, treating anemia early in renal failure has been demonstrated to slow the rate of decline of renal function [8].

Increasing evidence suggests that anemia in the diabetic population, whether type 1 or type 2, is a potent and independent predictor of increased risk for macrovascular and microvascular complications of diabetes. Despite these facts, anemia is unrecognized and largely untreated in patients with diabetes in Ethiopia particularly for populations in the study area. Therefore, the result of this study was aimed to identify the association of anemia and renal function test among DM patients attending Fenote Selam Hospital.

## Methods

### Study design and area

An Institutional-based cross-sectional study was conducted at Fenote Selam hospital from February 2012 to April 2012. The hospital consists of an operating room, one intensive care unit (ICUs) with 12 beds, 6 wards with 130 beds and an outpatient department. The study population was all DM patients who visit Fenote Selam Hospital, diabetes clinic during data collection time. Diabetic patients who have got anemia correction treatment like iron and transfusion therapy in the last three months of data collection were excluded from the study.

**Ethiopia** is a country located in the Horn of Africa. It is bordered by Eritrea to the north, Djibouti and Somalia to the east, Sudan and South Sudan to the west, and Kenya to the south. With over 91,000,000 inhabitants, Ethiopia is the most populous landlocked country in the world and the second-most populated nation on the African continent. It occupies a total area of 1,100,000 square kilometers (420,000 sq mi), and its capital and largest city is Addis Ababa.

**Finote Selam** is a town and separate woreda in western Ethiopia. Located in the Mirab Gojjam Zone of the Amhara Region, this town has a longitude and latitude of 10°42’N 37°16’E/10.7°N 37.267°E Coordinates: 10°42’N 37°16’E/10.7°N 37.267°E with an elevation of 1917 meters above sea level. In 1964, a hospital for lepers had been built in Finote Selam by the private fund “Swedish Aid to Leprous Children in Ethiopia”. The hospital is one of the zonal (large) hospital found in the Amhara region.

### Source population

The source population was all DM patients attending Fenote selam Hospital and the study population was all DM patients who visit Fenote Selam Hospital, diabetes clinic during data collection time. Out of which The DM patients who visit Fenote selam Hospital, diabetes clinic and become were our study participant.

### Sample size and sampling techniques

A total of 384 study participants were determined by single population proportion sample size calculation formula with study population size less than 10,000 (9). Systematic random sampling was used to select study participants. Every other study participants were selected after a random starting study participant who was selected by lottery method.

### Sample collection and examination

Socio demographic data were collected by using a structured questionnaire first prepared in English language and translated in to a local language Amharic and again translated to English. Two (2) ml venous blood was collected by vacutainer test tube coated EDTA anticoagulant for haemoglobin determination. The collected whole blood was mixed properly and analyzed for hemoglobin determination using CELL DYNE 1800 hematology analyzer. Five (5) ml venous blood was collected by vacutainer test tube without any anticoagulant for creatinine and Blood Urea Nitrogen determination. The whole blood without anticoagulant was allowed to clot for 15 to 30 minutes and was then centrifuged at 3000 rpm for 5 min and serum was separated. The separated serum was used to determine Creatinine and Blood Urea Nitrogen (BUN) using Humastar 80 chemistry analyzer.

### Data analysis

All the data were manually checked for its clarity and completeness and then coded and entered into Epi-info and transported to SPSS version 16.0 soft ware package for analysis. For controlling errors frequency checks was done. The data was analyzed for its descriptive statistics and bivariate logistic regression to determine the effect of various factors on the outcome variable and multivariate logistic regression to control confounding effect. The result was presented in the form of tables, figures and text using frequencies and summary statistics such as mean, standard deviation and percentage to describe the study population in relation to relevant variables. P-value less than 0.05 was taken as statistical significant.The degree of association between independent and dependent variables was assessed using odds ratio with 95% confidence interval of Bivariate and Multivariate logistic regression.

### Quality assurance

Quality assurance checks were performed daily according to the laboratory’s protocol. Commercial control materials was properly warmed and mixed according to the manufacturer’s recommendations. Internal quality control was conducted: after daily start up procedures are completed, when reagent lot changed, and when there is a reason to suspect an error in the data or results. Results was recorded, dated and initialed on the appropriate forms. Lot numbers, reagents, and expiration date are recorded where applicable.

### Ethical consideration

The study was reviewed and approved by the ethical committee of the School of Biomedical and Laboratory Sciences. Permission to conduct the study was also obtained from the Fenote selam hospital. Informed consent was obtained after each study participants was informed about the objective of the study and then any participant who is not willing to participate in the study was not forced to participate. They were also informed that all data obtained from them would be kept confidential by using codes instead of any personal identifiers and is meant only for the purpose of the study. Physicians were informed about Anemic and renal damaged patients for proper management.

## Results

### Socio-demographic characteristics of the diabetes mellitus patients

A total of 384 DM patients were involved in this study, of which 61.5% of the respondents were males and 38.5% were females. The mean age of participants was 40.96 with 16.8SD. Of the participants 226 (58.9%) were married, 66 (17.2%) were government employees and 347 (90.4%) were the followers of orthodox Christianity and 123 (32.0%) were unable to read and write. From the total DM patients, 166 (43.2%) were rural residents and 218 (56.8%) were urban residents (Table 1).

**Table 1:** Socio-demographic characteristics of DM patients attending fenote Selam Hospital, West Gojam, Northwest Ethiopia, 2012

**Variable (N = 384)**	**Total frequency**	**Percent (%)**
**Age groups**		
7-30	124	32.3
31-45	103	26.8
46-60	113	29.4
>60	44	11.8
**Sex**		
Male	236	61.5
Female	148	38.5
**Place of residence**		
Urban	218	56.8
Rural	166	43.2
**Educational status**		
Illiterates	123	32.0
Able to read & write	41	10.7
Primary educ. (1–8)	123	32.0
Secondary (9–10)	39	10.2
Preparatory(11–12)	32	8.3
College and above-	26	6.8
**Marital status**		
Single	94	24.5
Married	226	58.9
Divorced	18	4.7
Widowed	46	12.0
**Occupation**		
Students	48	12.5
Farmers	138	35.9
G. Employee	66	17.2
Merchants	34	6.9
Housewife	31	8.1
Others	67	17.4
**Religion**		
Orthodox	347	90.4
Others	37	9.6

### Anemia and clinical characteristics of diabetes mellitus patients

The distribution of type one and type two DM was 193(50.3%) and 191 (49.7%) respectively. The duration of DM with these patients ranges from one year to thirty years with the mean year of 5.87 and SD. 4.7 years. Seventy three (19.0%) of these DM patients was anemic (below 13 mg/dl for men and 12 mg/dl for women). Of these 38 (52.1%) and 35 (47.9%) were male and female respectively. Sixteen 16 (21.9%) and 57 (78.1%) of anemic DM patients were type one and type two respectively (Table 2).

**Table 2:** Anemia and clinical characteristics of diabetes mellitus patients attending fenote Selam Hospital, West Gojam, Northwest Ethiopia, 2012

	**Anemia**		
**Variables (N = 384)**	**Frequency (%)**	**Yes (%)**	**No (%)**
**Type of diabetes mellitus**			
Type 1	193 (50.3)	16 (4.2)	177 (46.1)
Type 2	191 (49.7)	57 (14.8)	134 (34.9)
**Duration with diabetes mellitus**			
<5 yrs	206 (53.6)	7 (1.8)	199 (51.8)
6-10	124 (32.6)	30 (7.8)	94 (24.5)
>11	54 (14.1)	36 (9.4)	18 (4.7)
**Type of treatment**			
Lente insulin	224 (58.3)	23 (6.0)	201(52.3)
Metformin	160 (41.7)	50 (13.0)	110 (28.6)
**Co-existing diseases**			
No	316 (82.3)	47 (12.2)	269 (70.1)
Yes	68 (17.7)	26 (6.8)	42 (10.9)
**Fasting blood sugar**			
<126	105 (27.3)	7 (1.8)	98 (25.5)
>126	279 (72.7)	66 (17.2)	213 (55.5)
**MCV**			
Normal	274 (71.4)	13 (3.4)	201 (68.0)
Decreased	110 (28.6)	60 (15.6)	50 (13.0)

### Renal function test results of diabetes mellitus patients

Urine Albumin, Serum Creatinine, Serum BUN, and Glomerular Filtration Rate (GFR) were determined for DM patients to determine the function of the kidney. Two hundred seventy three (71.1%) had a normal urine albumin where as 111 (28.9%) had increased urine albumin level. The mean estimated GFR was 112.12 ml / min/1.73 m^2^. Fifty three (13.8%), forty eight (12.5%), and two hundred eighty three (73.7%) of DM patients had an estimated GFR <60 ml/min/ 1.73 m, 60 – 90 ml/min/ 1.73 m, and > 90 ml/min/ 1.73 m, respectively (Table 3).

**Table 3:** Renal function test results among diabetes mellitus patients attending fenote Selam Hospital, West Gojam, Northwest Ethiopia, 2012

**Variables (N = 384)**	**Frequency**	**Percent (%)**
**Urine albumin**		
Normal	273	71.1
Increased	111	28.9
**Serum creatinine**		
Normal	295	76.8
Increased	89	23.2
**Serum BUN**		
Normal	291	75.8
Increased	93	24.2
**Glomerular filtration rate (GFR)**		
<60 ml/min/1.73 m	53	13.8
60 – 90 ml/min/1.73 m	48	12.5
>90 ml/min/1.73 m	283	73.7

### Bivariate risk factor analysis for anemia among diabetes mellitus patients

During bivariate analysis of variables, factors found to be significantly associated with anemia were: Age of the patients, Residence, Type of DM, Duration with Diabetes Mellitus, Type of treatment, Co-existing diseases, Fasting blood sugar (FBS), Mean cell volume, Urine Albumin, Serum Creatinine, Serum blood urea nitrogen (BUN), and Glomerular filtration rate (Table 4). Diabetes mellitus patients with an eGFR below 60 ml/min/ 1.73 m^2^ and between 60–89 ml/min/ 1.73 m^2^ have 11 times greater (AOR11.13, 95% CI =2.69, 45.94) and (AOR4.29 95% CI = 1.14, 16.13) risk to be anemic than DM patients with an eGFR above 90 ml/min/ 1.73 m^2^ respectively. Diabetes mellitus patients who have Type two DM have 4 times (COR = 4.71, 95% CI = 2.58, 8.55) greater risk to be than diabetes Mellitus patients who have Type one diabetes mellitus (Table 4).

**Table 4:** Bivariate logistic regression of selected variables in relation to anemia among diabetes mellitus patients attending fenote Selam Hospital, West Gojam, Northwest Ethiopia, 2012

	**Anemia**			
**Variables**	**Yes**	**No**	**AOR (95% CI)**	**P- value**
**Age groups**				
**7-30**	**9**	**115**	1*	
**31-45**	**9**	**94**	**1.22 (0.47, 3.21)**	**< 0.682**
**46-60**	**35**	**78**	5.73(2.61, 12.59)**	**< 0.001**
**>60**	**20**	**24**	10.65(4.32, 26.23)**	**< 0.001**
**Place of residence**				
**Urban**	**49**	**169**	1*	
**Rural**	**24**	**142**	0.58 (0.34, 0.99)**	**< 0.049**
**Educational status**				
**Illiterates**	**32**	**91**	1*	
**Able to read & write**	**14**	**27**	**1.47 (0.68, 3.16)**	**< 0.317**
**Primary educ. (1–8)**	**18**	**105**	0.48 (0.26, 0.93)**	**< 0.028**
**Secondary (9–10)**	**4**	**31**	0.33 (0.11, 0.98)**	**< 0.047**
**Preparatory(11–12)**	**3**	**29**	**0.29 (0.08,1.03)**	**< 0.056**
**College and above**	**2**	**24**	**0.24 (0.53, 1.06)**	**< 0.060**
**Marital status**				
**Single**	**9**	**85**	1*	
**Married**	**27**	**199**	**1.28 (0.58, 2.84)**	**< 0.541**
**Div. & Wid.**	**37**	**27**	12.94 (5.54, 30.20)**	**< 0.001**
**Occupation**				
**Students**	**7**	**41**	1*	
**Farmers**	**21**	**117**	**1.05 (0.42, 2.65)**	**< 0.916**
**G. Employee**	**15**	**51**	**1.72 (0.64, 4.62)**	**< 0.280**
**Merchants**	**10**	**24**	**2.44 (0.82, 7.25)**	**< 0.108**
**Housewife**	**4**	**27**	**0.86 (0.23, 3.25)**	**< 0.833**
**Others**	**16**	**51**	**1.84 (0.34, 4.89)**	**< 0.223**
**Type of diabetes mellitus**				
**Type 1**	**16**	**177**	1*	
**Type 2**	**57**	**134**	4.71 (2.58, 8.56)**	**< 0.001**
**Duration with diabetes mellitus**				
**<5 yrs**	**7**	**199**	1*	
**6-10**	**30**	**94**	9.07 (3.85, 21.41)**	**< 0.001**
**>11**	**36**	**18**	16.86(22.12,145.91)**	**< 0.001**
**FBS**				
**<126**	**7**	**98**	1*	
**>126**	**66**	**213**	4.34 (1.92, 9.80)**	**< 0.001**
**MCV**				
**Normal**	**13**	**201**	1*	
**Decreased**	**60**	**50**	14.09 (12.31, 47.16)**	**< 0.001**
**Urine albumin**				
**Normal**	**3**	**254**	1*	
**Increased**	**70**	**57**	19.93(18.61, 85.72)**	**< 0.001**
**Serum creatinine**				
**Normal**	**7**	**271**	1*	
**Increased**	**66**	**40**	11.06 (15.98, 60.36)**	**< 0.001**
**Serum BUN**				
**Normal**	**5**	**271**	1*	
**Increased**	**68**	**40**	11.95 (11.68, 41.27)**	**< 0.001**
**GFR**				
**< 60**	**51**	**6**	14.38 (18.23, 89.48)**	**< 0.001**
**60-90**	**20**	**25**	8.58 (10.21, 49.94)**	**< 0.001**
**>90**	**2**	**280**	1*	

*Reference category, ** significant association.

### Multivariate analysis of different variables relation to anemia among diabetes mellitus

All socio-demographic and other variables that showed significant associations and P-value < 0.2 with anemia in bivariate analysis were selected and entered for multivariate logistic regression analysis to identify the most important predictors of anemia. From the variables found to be significant in the bivariate analysis only Type of DM, Duration with DM, Mean cell volume, Glomerular filtration rate, Serum Creatinine, and marital status were found to be significantly associated with anaemia in multiple logistic regression analysis using P-value, odds ratio, and confidence interval. Diabetes mellitus patients with a glomerular filtration rate between 60–90 ml/min/ 1.73 m have a 4 times greater (AOR = 4.29, 95% CI = 1.14, 16.13) risk to be anemic than DM patients with a normal Glomerular Filtration Rate. Diabetes mellitus patients with a moderate renal failure had an 11times greater risk (AOR = 11.13, 95% CI = 2.69, 45.94) to be anemic than a normal renal function (Table 5).

**Table 5:** Multi-variate logistic regression of selected variables in relation to anemia among diabetes mellitus patients attending Fenote Selam Hospital, 2012

	**Anemia**			
**Variables**	**Yes**	**No**	**AOR (95% CI)**	**P- value**
**Type of diabetes mellitus**				< 0.001
Type 1	16	177	1*	
Type 2	57	134	4,71 (2.58, 8.56)**	
**Duration with diabetes mellitus**				<0.024
<5 yrs	7	199	1*	
6-10	30	94	4.38 (1.22,15.75)**	
>11	36	18	16.05 (3.92, 65.72)**	<0.001
**MCV**				<0.001
Normal	13	201	1*	
Decreased	60	50	10.47(3.51, 31.24)**	
**eGFR**				<0.001
<60	51	6	11.13(2.69, 45.94)**	
60-90	20	25	4.29 (1.14, 16.13)**	<0.031
>90	2	280	1*	
**Serum creatinine**				<0.001
Normal	7	271	1*	
Increased	66	40	8.55 (2.55, 28.62)**	
**Marital status**				<0.021
Single	9	85	1*	
Married	27	199	0.116 (0.019, 0.719)**	
Div. & Wido.	37	27	0.688 (0.109, 4.352)	<0.691

*Reference category, ** significant association.

## Discussion

Among 384 DM patients, 61.5% of the respondents were males and 38.5% were females, 50.3% were type one and 49.7% were type two DM. The current study showed that the overall prevalence of anemia in DM patients was 19%. This prevalence is in agreement with the studies done in other countries [9-11]. This over representation of males might be due to: the prevalence of DM is higher in males than females in the study area, mostly females prefer to go to Traditional healers to use traditional medicine than using hospitals and other health facilities and Most females were excluded due to iron therapy for anemia correction.

This study revealed that anemia is significant in Type two DM with AOR of 4.17 (95% CI = 2.58, 8.56) and anemia is also associated with duration of Diabetes Mellitus for greater than eleven years are seven times (AOR 7.47, 95% CI 1.51, 37.07) more likely to develop anemia than patients with DM for less than five years. This result is in agreement with other studies [12,13].

This study also showed that the prevalence of anaemia increases with older ages (27.4%) of DM patients whose age is greater than 60 years are anemic. This result is in agreement with a study in Israel, which indicates age has a significant association with anaemia in DM patients [14]. Although it was previously believed that declines in hemoglobin levels might be a normal consequence of aging, evidence has accumulated that anemia does reflect poor health and increased vulnerability to adverse outcomes in older persons.

The result of this study shows that 13.8% of the population with diabetes have clinically significant chronic kidney disease (CKD), as defined by an eGFR <60 and 60–89 ml/min/1.73 m2, respectively. This finding is in agreement with a study in USA which says 15.9% of participants had at least moderately reduced kidney function [15].

The prevalence of albuminuria in this study is 33.1%, of these 14.1% had moderate renal failure and 10.7% had mild renal failure. Sixty five percent of DM patients who have a normal albumin level in their urine had a normal renal function. This result is in agreement with a study in Israel which shows prevalence of elevated albuminuria (micro or macroalbuminuria) was 38.1%. 8.1% of patients had moderate and 31.4% had mild renal impairment [14].

The study results showed that there is a significant association between anaemia and renal function. Diabetes mellitus patients with a mild renal failure have 4 times greater (AOR = 4.29, 95% CI = 1.14, 16.13) risk to be anemic than DM patients with a normal renal function. As the glomerular filtration rate increase, the risk to be anemic will decrease dramatically. Diabetes mellitus patients with a moderate renal failure have 11 times greater (AOR = 11.13, 95% CI = 2.69, 45.94) risk to be anemic than a normal renal function. This finding is in agreement with a study in UK, which indicates from anemic patients 36% with moderate renal failure and 9% of those with mild renal failure [10].

In this study, the relation between HGB and eGFR became negatively linear, as the estimated glomerular filtration rate decreases, the level of haemoglobin increases. The result of this study is in line with a retrospective study in UK, the prevalence of anemia increased progressively with worsening CKD. People with CKD stage 3 accounted for the largest number of people with anemia; 18% (95% CI13–24) had HGB < 110 g/l. Most patients with diabetes and anaemia can be identified by examining patients with moderate to severe renal impairment. These strong links between the kidney failure and anaemia in diabetes probably reflect the unique vulnerability of the renal microcirculation to damage in diabetes [16].

Renal function as measured by eGFR was the strongest predictor of anemia. Additional factors present in diabetes mellitus patients may contribute to the development of increased risk for anemia in patients with diabetes. These factors are: Age of patients, Type of DM, and duration with DM. The strengthen of this study is that it is one of few studies in developing countries where chronic disease like DM becoming more common but the study is not without limitations and the limitations of this study are lacks control groups, one time GFR is measured only one time which may not tell us the details of renal problem, as well proteinuria is measured only once this may be affected by different factors at that spot rather than being pathological condition and small sample size used for the study and which is not able to generalize to the whole population of Ethiopia and inclusion of young age of Participants may also affect the result.

## Conclusion

This study showed that anemia is public health problem in the DM patients in the study area. As well this study showed that there is a significant association between anaemia and renal function. The prevalence of anaemia increases as kidney function declines. Patients at greatest risk can be identified by the presence of renal disease i.e. by measuring their level of estimated glomerular filtration rate (eGFR). Therefore, DM patients should be strictly monitored for renal failure and anemia for proper management of diabetes patients as well physicians giving care for DM patients should investigate the presence of anemia and renal failure and treat as a routine work.

## Competing interests

The authors declare that they have no competing interests.

## Authors’ contributions

AA: initiated the study and made major xcontributions to the study design and statistical analysis. WB: conceived the study, undertook statistical analysis and drafted the manuscript. AbA: initiated the study, undertook statistical analysis and has major contribution in drafting the manuscript. All authors contributed to the writing of the manuscript and approved the submitted version of the manuscript.

## Pre-publication history

The pre-publication history for this paper can be accessed here:

http://www.biomedcentral.com/2052-1839/13/6/prepub
